# Mr. Collin's Case of Diseased Bladder

**Published:** 1807-02

**Authors:** John Charles Collins

**Affiliations:** Member of the College of Surgeons, Physical Society at Guy's and Royal Medical Society, Edinburgh. Swansea


### To Dr. BRADLEY.

Sir,

I Beg you will insert the following Case in the next Number of the Medical Journal if received in due time.

I am, Sir, yours, &c.

*Swansea, Jan.* 8, 1807. JOHN CHARLES COLLINS,

Member of the College of Surgeons, Physical Society at Guy's, and Royal Medical Society, Edinburgh.

---

Capt. L. Dalton, aged 25, of a dark melancholic temperament, complained of great pain, extending from the lumbar vertebræ almost to the umbilicus, with an evident tumefaction and hardness at the part. The sense of pain sometimes confined to a small spot, at others more general; also pain in his shoulders, particularly the right; sometimes in the chest, with difficulty of breathing; his bowels costive; urine at times depositing a white purulent sediment, not always, but affording relief for a day or two by the discharge; no pain in making it, but latterly a shooting pain down the right extremity and testicle: says he has had the complaint for some months; attributed it to a strain, occasioning great weakness in his back, with difficulty of voiding the urine; when without the purulent discharge perfectly natural.—Countenance sallow; tongue furred; thirst at times considerable; no shivering during our attendance, with head-ach. From these symptoms, and a knowledge of the habit of the patient, I concluded that his liver was affected, and that the matter might have been taken up and discharged by urine, for which I ordered calomel with a spare diet, and the local application of mercurial plaster; but getting worse, he sent for Dr. Turton, who, upon examination, seemed to think that there might

 be be a schirrous affection of his kidney, pancreas, or some of the mesenteric glands, consequently he ordered some drastic purges, as gamboge, with a stimulating diet. The purgatives evidently relieved him for some time, and they were changed for the uva ursi and bark; but in a few days the patient growing worse, Dr. Elliot was requested to attend; and upon a consultation, it seemed probable that there was a complicated disorder of the liver and kidney. As the diseased tumour was evidently in that region which the liver fills, and the matter as evidently came from the kidney, and that only from one, the quantity of natural urine being very considerable, in addition to what came with the matter, calomel with saline medicines, occasional purgatives, and the local application of mercury, blisters, &c. which produced mere temporary relief, and at the expiration of three months from his first complaining to me, he died; but before that happened, he acknowledged having received a very considerable blow on the part affected, about two years since. The disease being most certainly of a very obscure nature, I was happy in having an opportunity of examining it, and I shall now briefly state the appearances upon dissection.

The peritoneum was discoloured, as if by inflammation; liver of a natural colour upon its anterior surface, but very dark upon its posterior; several adhesions to the peritoneum; stomach contained the liquid food given just before death; omentum *inflamed*, as well as all the intestines, which seemed to have a considerable quantity of fæces in them; gall-bladder with a little dark coloured bile.

Right kidney apparently enveloped in a large sac, containing about three pints of matter; the ureters enlarged, and this tumour projecting with the kidney anteriorly to the edge of the ribs, consequently pressing the liver downwards.

The pancreas, spleen, and mesenteric glands all sound; the left kidney very much enlarged, without any active disease, but flaccid.

Bladder sound, with a good deal of urine in it. On opening the thorax, the lungs evidently inflamed, with partial spots.

The heart completely attached to the pericardium, without the least separation, consequently no liquor, and the muscular fibres extremely flaccid and soft, that it might almost be said to be no heart, so little had it the appearance of one.

Upon more minute inspection of the right kidney, the whole whole internal substance, both medullary as well as cortical, seemed destroyed, and nothing but the membranous covering remaining; attached to which, and opening into the ureter, the great sac with the pus was found. It appeared that the matter formed in the kidney was discharged into this sac, evidently formed by an enlargement of the pelvis of the kidney, and thus accounting for the occasional discharge of matter. If it had not been for this, the matter must have constantly flowed into the bladder, and no natural urine passed.

